# Whole-Inactivated Influenza Virus Is a Potent Adjuvant for Influenza Peptides Containing CD8^+^ T Cell Epitopes

**DOI:** 10.3389/fimmu.2018.00525

**Published:** 2018-03-14

**Authors:** Peter C. Soema, Sietske K. Rosendahl Huber, Geert-Jan Willems, Ronald Jacobi, Marion Hendriks, Ernst Soethout, Wim Jiskoot, Jørgen de Jonge, Josine van Beek, Gideon F. A. Kersten, Jean-Pierre Amorij

**Affiliations:** ^1^Intravacc (Institute for Translational Vaccinology), Bilthoven, Netherlands; ^2^Division of Drug Delivery Technology, Cluster BioTherapeutics, Leiden Academic Centre for Drug Research (LACDR), Leiden University, Leiden, Netherlands; ^3^Centre for Infectious Disease Control Netherlands, National Institute for Public Health and the Environment (RIVM), Bilthoven, Netherlands

**Keywords:** whole-inactivated influenza virus, adjuvant, T cell peptide, CTL, design of experiments, formulation, peptide vaccine

## Abstract

Influenza peptide antigens coding for conserved T cell epitopes have the capacity to induce cross-protective influenza-specific immunity. Short peptide antigens used as a vaccine, however, often show poor immunogenicity. In this study, we demonstrate that whole-inactivated influenza virus (WIV) acts as an adjuvant for influenza peptide antigens, as shown by the induction of peptide-specific CD8^+^ T cells in HLA-A2.1 transgenic mice upon vaccination with the influenza-M1-derived GILGFVFTL peptide (GIL), formulated with WIV. By screening various concentrations of GIL and WIV, we found that both components contributed to the GIL-specific T cell response. Whereas co-localization of the peptide antigen and WIV adjuvant was found to be important, neither physical association between peptide and WIV nor fusogenic activity of WIV were relevant for the adjuvant effect of WIV. We furthermore show that WIV may adjuvate T cell responses to a variety of peptides, using pools of either conserved wild-type influenza peptides or chemically altered peptide ligands. This study shows the potential of WIV as an adjuvant for influenza peptides. The simple formulation process and the solid safety record of WIV make this an attractive adjuvant for T cell peptides, and may also be used for non-influenza antigens.

## Introduction

Current seasonal influenza vaccines exert their protective effect mainly through the induction of virus-specific neutralizing antibodies directed against the surface proteins of influenza virus (1). However, changes in the influenza virus surface proteins caused by antigenic drift or shift allow influenza viruses to evade these antibodies. Therefore, influenza vaccines require regular updates or need to be completely renewed, to be able to protect against circulating influenza viruses.

By contrast, cellular immune components such as CD8^+^ and CD4^+^ T cells often recognize conserved epitopes of internal influenza virus proteins. This allows these T cells to cross-react with various influenza strains or even subtypes, and provides the host with a better protection against drifting or shifting influenza virus strains (2–4). Cytotoxic CD8^+^ T cells (CTLs) can clear virus-infected host cells, thereby controlling influenza virus infections by inhibiting viral replication and potentially limiting viral spread. The induction of influenza virus-specific T cells in addition to neutralizing antibodies may, therefore, increase the effectiveness of influenza vaccines. Currently, multiple approaches for the induction of influenza-specific T cell responses by vaccination are being investigated (5, 6).

Short linear peptides that represent conserved T cell epitopes can be used as antigens to induce influenza virus-specific CTLs (6). However, peptides are generally not that immunogenic, as they are inefficiently delivered to antigen-presenting cells (APCs), lack pathogen-associated molecular patterns (PAMPs) to trigger the immune system, and are rapidly degraded. Inefficient delivery of an antigenic peptide and lack of PAMPs can be overcome by formulation of the peptide with an appropriate adjuvant (7).

Water-in-oil emulsions such as incomplete Freund’s adjuvant (IFA) are effective adjuvants for peptides, but are associated with substantial adverse events such as lesion formation at the site of injection, making them undesirable for use in humans for vaccination (6). Thus, alternative adjuvants for peptide antigens are highly sought after.

Whole-inactivated influenza virus (WIV) possesses an innate adjuvant capability in the form of viral single-stranded RNA (ssRNA), which is a potent TLR7 agonist (8). Furthermore, WIV contains CD4^+^ epitopes, which are favorable for the induction of functional CD8^+^ T cell and B cell responses (9). WIV is also more reactogenic than split and subunit influenza vaccines, which could be linked to its increased immunogenicity. A recent study by Babb et al. described that gamma-irradiated WIV can act as an adjuvant for a Semliki Forest virus (SFV) vaccine, which significantly increased the induction of SFV-specific antibodies in mice (10). However, it has not been studied whether WIV can be used as an adjuvant for T cell-inducing peptide-based vaccines.

Another method to increase the immunogenicity of peptides is to increase their binding affinity to MHC class I molecules by chemical modification. These so-called, chemically altered peptide ligands (CPLs) derived from HLA-A2.1-restricted epitopes were shown to possess a higher affinity to HLA-A2.1, and induced more IFN-γ positive splenocytes compared to wild-type (WT) epitopes in HLA-A2.1 transgenic mice (11). Chemical modification of subdominant peptide epitopes might, therefore, increase the breadth of peptide-specific T cell responses.

In the current study, first we investigated the effect of the addition of WIV to the GILGFVFTL (GIL, M1_58–66_) peptide, which is the dominant CD8^+^ T cell epitope in HLA-A2.1-restricted individuals. Next, we performed a dose-finding study to determine the optimal WIV and peptide antigen concentrations for the induction of peptide-specific T cells. Furthermore, we studied the effect of WIV-peptide co-localization, association, and WIV membrane fusion activity on the adjuvant activity of WIV. Finally, we investigated the adjuvant activity of WIV on influenza peptides coding for conserved WT T cell epitopes and CPL variants.

## Materials and Methods

### Formulation of Vaccines

Influenza A/PR/8/34 (H1N1) virus was propagated on fertilized eggs and inactivated with β-propiolactone as described before (12, 13), which yielded PR8 WIV bulk vaccine. For certain experiments, WIV was subsequently fusion-inactivated by lowering the buffer pH to 4.5 for 15 min at 37°C with a pre-titrated volume of 1 M HCl (Sigma-Aldrich) and was then brought back to physiological pH by dialyzing overnight against PBS pH 7.2 (Life Technologies). Membrane fusion capacity was subsequently determined by a hemolysis assay as described previously (14).

The Netherlands Cancer Institute kindly provided the HLA-A2.1-restricted influenza GILGFVFTL (GIL, M1_58–66_), FMYSDFHFI (FMY, PA_46–54_), and NMLSTVLGV (NML, PB1_413–421_) peptides, and chemically altered CPLs [am-phg]ILGFVFTL (G1), [4-FPHE]MYSDFHF[2-AOC] (F5), and N[NLE]LSTVLGV (N53), as published by Rosendahl Huber et al. (11) (Figure S1 in Supplementary Material).

Influenza PR8 WIV and peptide antigens were formulated in PBS pH 7.2 at various concentrations (concentration of WIV is total protein content). Where mentioned, 50 µg of CpG ODN 1826 (Invivogen) or 50% (v/v) IFA (Sigma-Aldrich) were added to the formulation.

### Determination of Association Between Peptides and WIV

The association of peptides to WIV particles was studied by quantification of unassociated peptide in a mixture of peptides and WIV. Peptides were mixed with WIV in similar concentrations as used in the animal studies. WIV particles were subsequently spun down by ultracentrifugation for 2 h at 30,000 × *g*. Supernatant was collected and analyzed for peptides by mass spectrometry on a nanoscale LC-MS system, essentially as described by Meiring et al. (15), comprising a 100- and 50-µm internal diameter in house-packed Reprosil-Pur C18-AQ trapping and analytical column, respectively. Peptides were trapped in 100% of solvent A (water + 5% dimethylsufoxide + 0.1% formic acid) for 10 min. The linear gradient for the separation ranged from 15 to 90% of solvent B (acetonitrile + 5% DMSO + 0.1% FA) in 25 min at a flow rate of 125 nL/min. The column effluent was directly electro-sprayed into the MS using a gold-coated fused silica-tapered tip of 3.5 µm internal diameter. High-resolution MS1 data were acquired on an LTQ-Orbitrap XL mass spectrometer (Thermo Scientific, San Jose, CA, USA) at a resolution of 60,000 FWHM. Peptide identity was confirmed by their CID MS/MS fragmentation spectra, acquired on-the-fly in the LTQ mass analyzer on the singly or doubly charged ions of the targeted peptides only. The percentage of unassociated peptide was calculated by comparing peptide content in supernatants of peptide mixed with WIV to peptide content in supernatants collected from solutions without WIV.

### Hemolysis Assay

Virosome fusion activity was determined by using a hemolysis assay as described previously (16). Formulations were mixed with human blood erythrocytes and 0.1 M 2-(N-morpholino)ethanesulfonic acid (MES) buffer with pH ranging from 4.5 to 5.5, and incubated at 37°C for 30 min. The released hemoglobin was quantified in the supernatant after centrifugation by reading absorbance at 540 nm using a Synergy Mx reader (Biotek). Hemoglobin release from erythrocytes mixed with water was set as maximal hemolysis (100%).

### Animal Studies

Animal studies were conducted according to the guidelines provided by the Dutch Animal Protection Act and were approved by the Committee for Animal Experimentation (DEC) of the PD-Alt campus (Bilthoven, The Netherlands) under protocol number PO201400134. Eight- to ten-week-old female transgenic C57BL/6-Tg(HLA-A2.1)1Enge/J mice (Jackson Laboratory, maintained in-house) were used in all studies.

In the proof-of-principle study, mice (three per group) received immunizations subcutaneously (s.c.) in alternating flanks at days 0 and 21, containing either PBS, 50 µg WIV, 1 nmol GIL peptide adjuvanted with 50 µg WIV or 100 nmol GIL adjuvanted with 50 µg CpG in a volume of 100 µL.

For the dose-finding study, formulations consisting of various doses of WIV and GIL peptide were administered s.c. in alternating flanks of mice (six per group) at days 0 and 21.

To study the effect of adjuvant co-localization, mice (six per group) were immunized at days 0 and 21 either s.c. in one flank with PBS or 100 nmol GIL peptide adjuvanted with 25 µg WIV, or s.c. in separate flanks with 100 nmol GIL peptide in one flank and 25 µg WIV adjuvant in the opposite flank.

The effect of membrane fusion activity was assessed by immunizing mice (six per group) s.c. in alternating flanks at days 0 and 21 with 100 nmol GIL peptide adjuvanted with either 25 µg of fusion-active WIV or fusion-inactive WIV.

The adjuvant effect of WIV on a mix of multiple peptides was assessed with either a WT peptide pool (GIL, FMY, and NML; 100 nmol each) or a modified peptide pool (G1, F5, and N53; 100 nmol each). Mice (six per group) received an s.c. immunization in the flank at days 0 and 21 containing either PBS, WT peptide pool adjuvanted with 5 µg WIV or IFA, CPL peptide pool adjuvanted with 5 µg WIV or IFA, or only 5 µg WIV. In all studies, animals were sacrificed at day 35.

### Intracellular Staining and Flow Cytometry

T cell populations were analyzed by flow cytometry. In short, single-cell suspensions of splenocytes were plated at a concentration of 2×10^6^ cells in a 48-well plate in RPMI medium (Life Technologies) with 10% Hyclone fetal calf serum (FCS, Thermo Scientific), and stimulated overnight with either medium, 50 ng peptide or 50 ng PR8 WIV. Cytokine transport was blocked by incubating with Golgi-plug (BD Biosciences) for the last 4 h. Cells were stained with anti-mouse CD8-FITC (BD Biosciences), anti-mouse CD4-PE (BD Biosciences), and Live-dead-Aqua (Invitrogen), fixated with fixation/permeabilization buffer (BD Biosciences), and washed with permeabilization wash buffer (BD Biosciences). Finally, cells were stained intracellularly with anti-mouse IFN-γ-APC (BD Biosciences), and IFN-γ^+^ CD8^+^ T cells were quantified on a FACS Canto II flow cytometer (BD Biosciences). Acquired data were analyzed with FlowJo version 10 for Mac OSX (TreeStar Inc.). Gating strategy for the quantification of CD8^+^ IFN-γ^+^ T cells is shown in Figure S2 in Supplementary Material.

### Enzyme-Linked Immunosorbent Spot Assay (ELISpot)

Peptide-specific, IFN-γ-producing T cells were determined in splenocytes by an IFN-γ ELISpot. 96-wells Multiscreen PVDF filter plates (Millipore) were activated by adding 25 µL 70% ethanol for 2 min and washed three times with PBS. Plates were coated overnight with anti-mouse IFN-γ antibodies (U-Cytech) at 4°C, washed three times, and blocked with 5% FCS (Hyclone, Thermo Scientific) in RPMI medium for 1 h at 37°C. Subsequently, 4×10^5^ isolated splenocytes resuspended in RPMI medium, 5% FCS was added to each well with or without 100 ng of relevant peptide, and incubated overnight at 37°C, 5% CO_2_. Next, filter plates were washed five times and IFN-γ was detected using biotinylated anti-mouse antibodies (U-Cytech) and 100 µL BCIP/NBT reagent (Thermo Scientific) per well. Plates were washed with tap water and dried. Spots were counted using an A.EL.VIS ELISpot reader (Aelvis). The number of IFN-γ-producing cells in antigen-stimulated splenocytes was counted and corrected for background by subtracting the number of spots produced by splenocytes incubated with medium only.

### Statistics

Results were statistically analyzed with a one-way ANOVA followed by a Tukey post test for multiple comparisons. All statistical analyzes were performed using GraphPad Prism 6.04 for Windows (GraphPad Software Inc.).

## Results

### Addition of WIV to GIL Peptide Enhances GIL-Specific T Cell Responses

**Figure 1:**
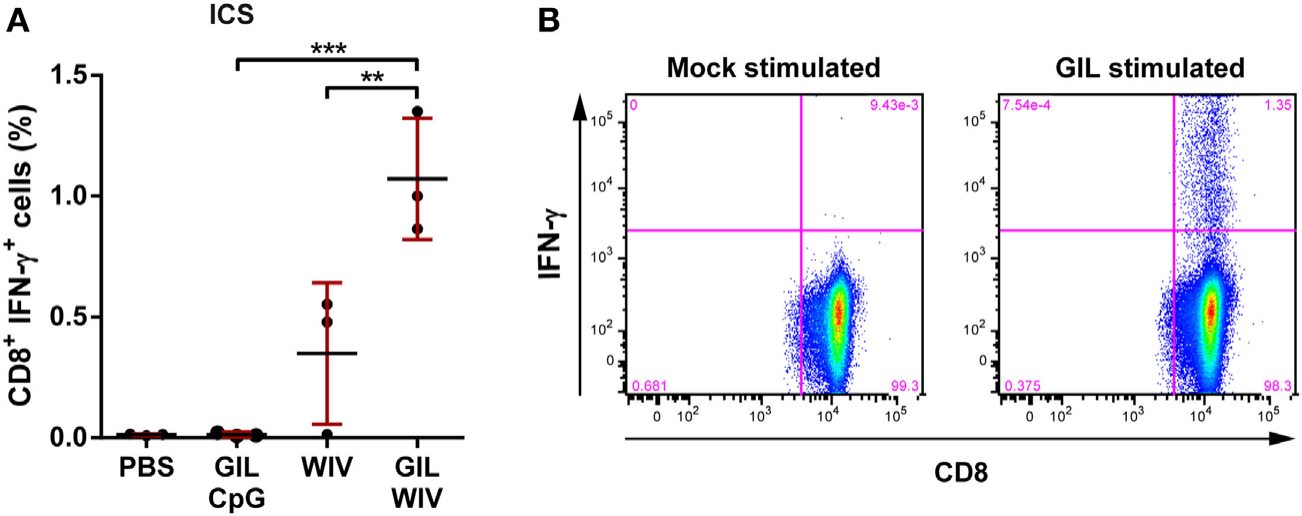
Comparison of CD8^+^ T cell responses induced by 1 nmol GIL peptide adjuvanted with either 50 µg CpG or 50 µg whole-inactivated influenza virus (WIV). HLA-A2.1 transgenic mice were injected twice, 3 weeks apart with PBS (negative control), WIV or GIL peptide with indicated adjuvant. Shown are the responses 2 weeks after the final immunization. **(A)** Splenocytes were re-stimulated with GIL peptide and stained for intracellular cytokine IFN-γ. The percentages for IFN-γ^+^ CD8^+^ T cells acquired by FACS are plotted. Data are shown as mean ± SD of three mice per group, each circle is a single replicate and data are from a single experiment representative of two individual experiments. **p < 0.01, ***p < 0.001 (one-way ANOVA). **(B)** Representative FACS plot displaying GIL-specificity of IFN-γ^+^ CD8^+^ T cells in splenocytes from mice immunized with GIL peptide formulated with WIV and re-stimulated with GIL peptide.

As shown previously, 100 nmol GIL peptide, without adjuvant, was unable to induce detectable GIL-specific T cell responses in HLA-A2.1 transgenic mice (14). The addition of the adjuvant CpG, a TLR9 agonist, was also insufficient to induce GIL-specific CD8^+^ T cells (Figure 1). By contrast, the addition of WIV to the GIL peptide resulted in the induction of high levels of GIL-specific CD8^+^ T cell responses. Importantly, WIV alone was able to induce low levels of GIL-specific CD8^+^ T cells, which is caused by processing of the GIL epitope present in the internal M1 protein of WIV. The combination of GIL and WIV, however, induced high levels of GIL-specific T cells as compared to WIV only.

### Both GIL and WIV Contribute to the Induction of GIL-Specific T Cell Responses

To investigate the relative contributions of WIV on the induction of GIL-specific T cell responses, different combinations of amounts of peptide and WIV were tested in a mouse model by determining IFN-γ responses with FACS and ELISpot analyses. The peptide antigen dose ranged from 1 to 100 nmol, and the WIV dose ranged from 1 to 25 µg.

**Figure 2:**
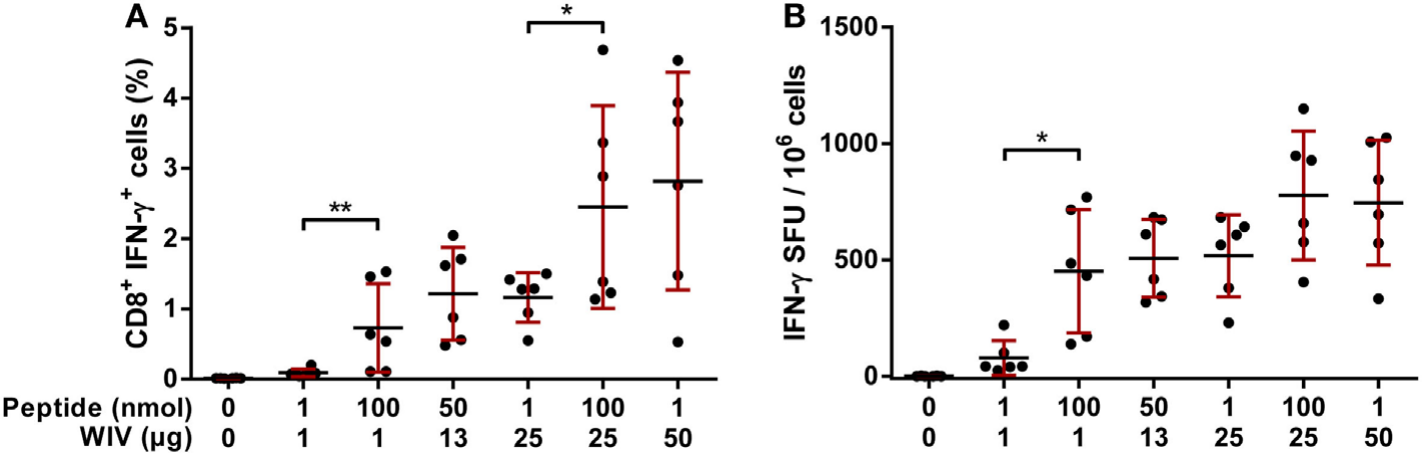
Induction of GIL-specific T cell responses by different concentrations of GIL and whole-inactivated influenza virus (WIV). HLA-A2.1 transgenic mice were vaccinated twice, 3 weeks apart with indicated amounts of GIL peptide and WIV. **(A)** Splenocytes were re-stimulated with GIL peptide and stained for intracellular cytokine IFN-γ. The percentages for IFN-γ^+^ CD8^+^ T cells acquired by FACS are plotted. **(B)** Splenocytes of immunized mice were re-stimulated with GIL peptide. The number of CD8^+^ IFN-γ-secreting cells was subsequently determined with ELISpot. **(A,B)** Data are shown as mean ± SD of three mice per group, each dot is a single replicate and data are from a single experiment. *p < 0.05, **p < 0.01 (one-way ANOVA).

A combination of 1 nmol GIL peptide and 1 µg WIV induced very low responses (Figures 2A,B). Increasing the GIL dose to 100 nmol led to a significant induction of GIL-specific CD8^+^ T cells. This effect was also observed when the peptide dose was increased from 1 to 100 nmol combined with a dose of 25 µg WIV. The increase of WIV from 1 to 25 µg also had a marked effect on the induction T cells, both with 1 and 100 nmol GIL. These data show that GIL and WIV induce the highest T cell responses when combined, whereas GIL or WIV alone induce considerably less T cells. It should be noted that it is estimated that 25 µg WIV contains approximately 535 pmol of GIL peptide; a negligible amount compared to the 1–100 nmol GIL peptide. These findings imply that the addition of WIV provides a synergistic, adjuvant-like effect for the GIL peptide.

### Co-Localization of GIL and WIV Is Necessary to Obtain the Adjuvant Effect of WIV

**Figure 3:**
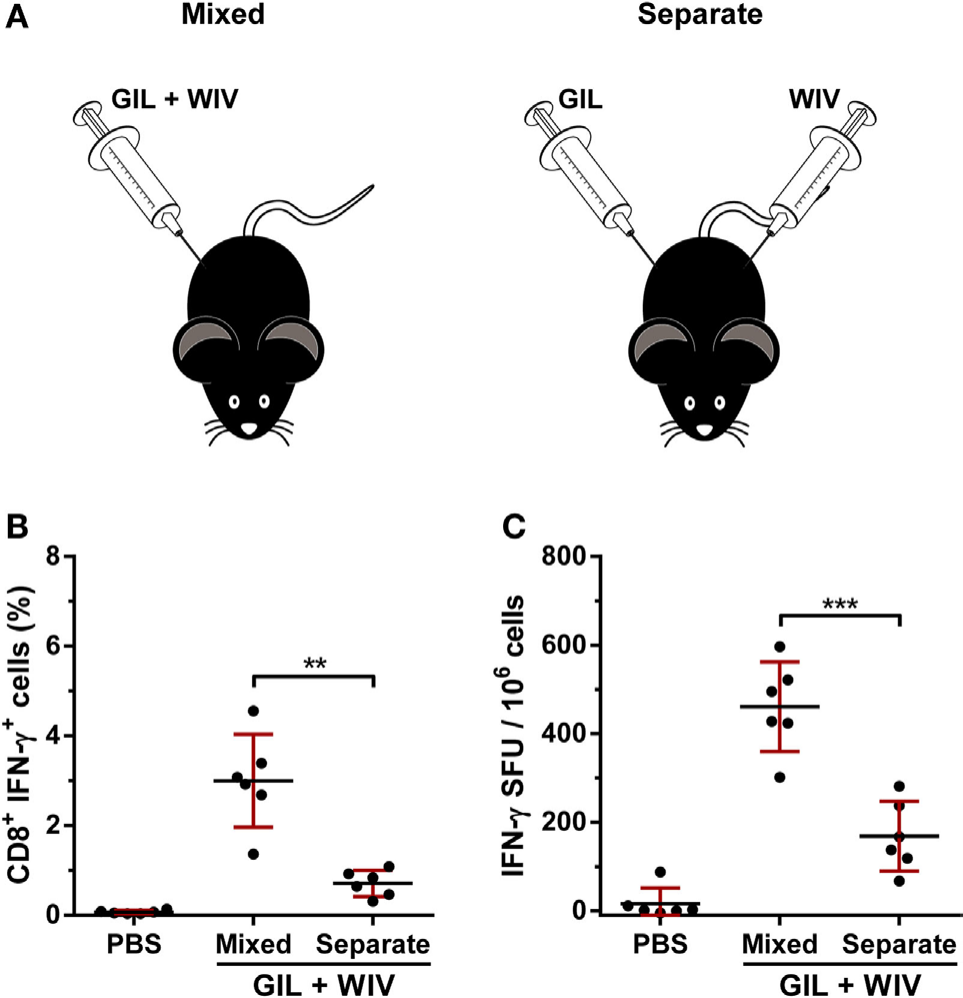
Influence of co-localization on the immunogenicity of GIL (100 nmol) and whole-inactivated influenza virus (WIV, 25 µg). **(A)** HLA-A2.1 transgenic mice were vaccinated twice, 3 weeks apart with GIL peptide and WIV either combined in one single flank (mixed) or separate in opposite flanks (separate). **(B)** Splenocytes were re-stimulated with GIL peptide and stained for intracellular cytokine IFN-γ. The percentages for IFN-γ^+^ CD8^+^ T cells acquired by FACS are plotted. **(C)** Splenocytes were re-stimulated with GIL peptide. The number of IFN-γ-secreting cells was subsequently determined with ELISpot. **(A,B)** Data are shown as mean ± SD of six mice per group, each dot is a single replicate and data are from a single experiment. **p < 0.01, ***p < 0.001 (one-way ANOVA).

The previous results show that, aside from providing additional GIL-epitopes, WIV acts as an adjuvant when combined with GIL. Next, the influence of co-localization of GIL (100 nmol) and WIV (25 µg) was investigated. When GIL and WIV were injected separately at two different sites (Figure 3A), only moderate GIL-specific T cell responses were obtained. By contrast, when injected at the same site, a significant induction of T cell responses was observed (Figures 3B,C), proving that the T cell response is not simply a sum of the amount of GIL present in the formulations. Thus, co-localization is necessary to obtain the adjuvant effect of WIV for the GIL peptide.

### The Adjuvant Effect of WIV Is Not Caused by Physical GIL Peptide–WIV Interaction

For some adjuvants, direct physical association (such as adsorption) between adjuvant and antigen is required for optimal antigen delivery to APCs (14, 17). The adjuvant effect observed by WIV might also rely on physical association between WIV and GIL peptide, since co-localization was shown to be required. The association between GIL and WIV was, therefore, determined by using mass spectrometry after separation of non-associated peptide from WIV by centrifugation (Table 1). Overall, little to no association was found between GIL peptide and WIV at various concentrations. Some association was observed with higher concentrations of WIV (25–50 µg), but not at 1 µg WIV. However, no direct correlation can be seen between peptide–WIV association and the immunogenicity of the mixtures, indicating that physical interaction between GIL and WIV is not the predominant mechanism behind the adjuvant activity of WIV.

**Table 1:** Association between GIL peptide and whole-inactivated influenza virus (WIV).

GIL peptide (μg)	WIV (μg)	Unassociated GIL peptide (%)
1	1	112 ± 10
100	1	111 ± 6
50	13	96 ± 5
1	25	77 ± 8
100	25	92 ± 20
1	50	87 ± 9

*Indicated amounts of GIL peptide and WIV were mixed and subsequently separated by ultracentrifugation. The residual, unassociated peptide in the supernatant was quantified by using mass spectrometry. Data are shown as mean ± SD from three individual experiments*.

### Membrane Fusion Activity Is Not Required for WIV Adjuvant Activity

**Figure 4:**
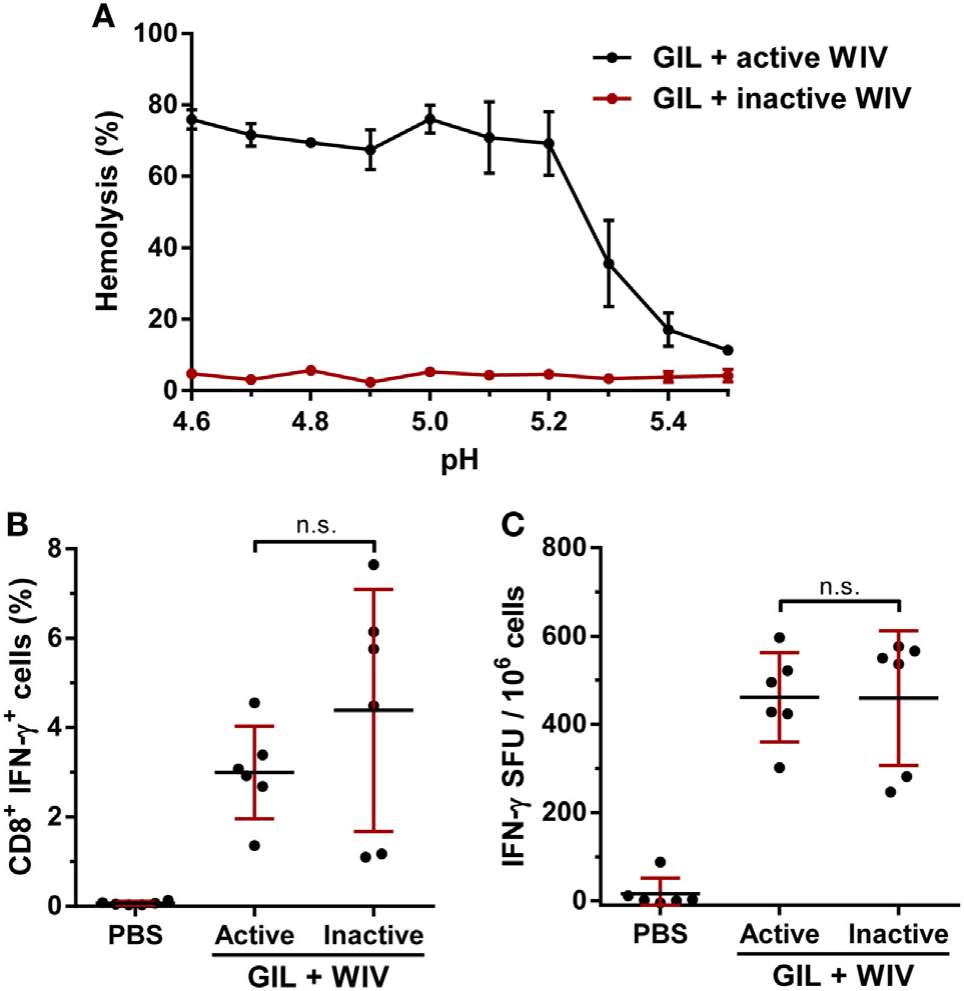
Effect of membrane fusion activity on whole-inactivated influenza virus (WIV) adjuvant activity. **(A)** WIV was fusion-inactivated by brief exposure to a buffer with pH 4.5 at 37°C. Hemolysis was performed by mixing either WIV (“active” WIV) or fusion-inactivated WIV (“inactive” WIV) with human blood erythrocytes at various pHs. Fusion-mediated hemoglobin release was subsequently determined by spectrophotometric analysis. Data are shown as mean ± SD of three individual experiments. **(B)** HLA-A2.1 transgenic mice were vaccinated twice, 3 weeks apart with GIL peptide (100 µg) and either fusion-active or fusion-inactive WIV (25 µg). Splenocytes were re-stimulated with GIL peptide and stained for intracellular cytokine IFN-γ. The percentages for IFN-γ^+^ CD8^+^ T cells acquired by FACS are plotted. **(C)** Splenocytes were re-stimulated with GIL peptide. The number of IFN-γ-secreting cells was subsequently determined with ELISpot. **(B,C)** Data are shown as mean ± SD of six mice per group, each dot is a single replicate and data are from a single experiment. n.s. = not significant (one-way ANOVA).

Budimir et al. have shown that the membrane fusion activity of WIV was necessary for the induction of cross-reactive T cell responses by WIV (18). Therefore, we investigated the influence of the membrane fusion activity of WIV on the immunogenicity of the GIL and WIV combination. WIV was fusion inactivated by exposure to acidic pH, and loss of membrane fusion activity was confirmed by a hemolysis assay (Figure 4A). A mixture of GIL peptide and fusion-inactivated WIV was able to induce peptide-specific T cell responses similar to those induced by GIL peptide with fusion-active WIV (Figures 4B,C), indicating that fusion activity is not critical for the adjuvant activity of WIV.

### WIV Can Act as an Adjuvant for Multiple Combined Peptides

To increase the efficacy and broadness of the peptide-induced immune response, multiple conserved epitopes should be included into a peptide-based influenza vaccine. To investigate whether WIV also acts as an adjuvant for other peptides, two subdominant conserved human HLA-A2.1-restricted influenza epitopes, FMYSDFHFI (FMY, PA_46–54_) and NMLSTVLGV (NML, PB1_413–421_), were added to the mixture of GIL and WIV. Another method to improve the immunogenicity of peptide antigens is by increasing the binding affinity to MHC I through chemical modification (11, 19). To examine whether this in combination with the improved immunogenicity of WIV adjuvant would further boost the immune response, chemically altered variants of the GIL, FMY, and NML peptide ligands, being [am-phg]ILGFVFTL (G1), [4-FPHE]MYSDFHF[2-AOC] (F5), and N[NLE]LSTVLGV (N53), were combined with WIV. As a reference control, peptides were also adjuvanted with the water-in-oil-based IFA.

**Figure 5:**
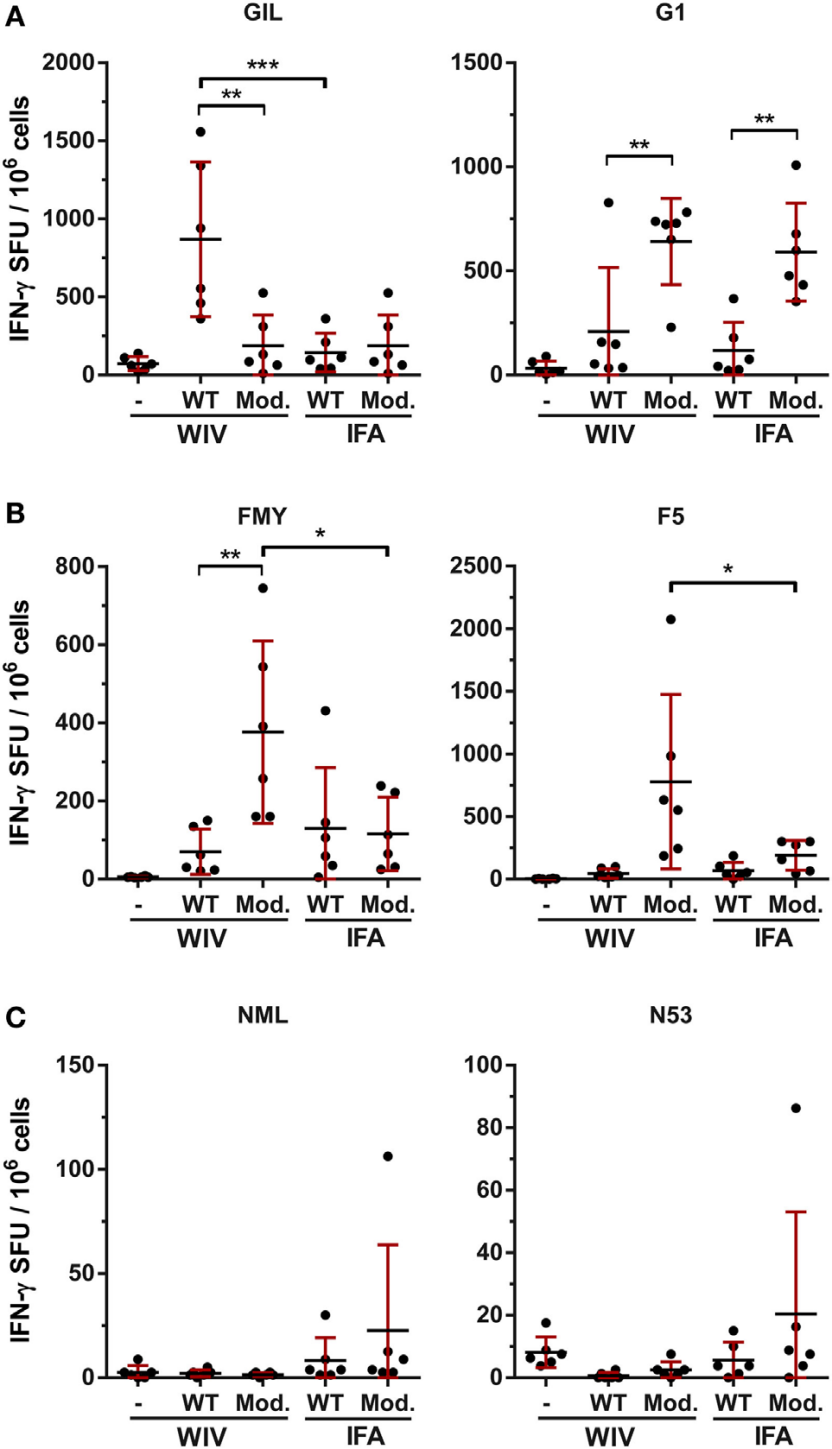
T cell responses against wild-type and modified peptides adjuvanted with whole-inactivated influenza virus (WIV). HLA-A2.1 transgenic mice were vaccinated twice, 3 weeks apart with peptide pools containing either wild-type (WT) peptides (GIL, FMY, and NML, 100 nmol each) or modified (mod.) peptides (G1, F5, and N53, 100 nmol each) adjuvanted with either WIV or 50% (v/v) incomplete Freund’s adjuvant (IFA). **(A)** Splenocytes of immunized mice were re-stimulated with either GIL or G1 peptide. **(B)** Splenocytes were re-stimulated with either FMY or F5 peptide. **(C)** Splenocytes were re-stimulated with either NML or N53 peptide. **(A–C)** The number of IFN-γ-secreting cells was subsequently determined with ELISpot. Data are shown as mean ± SD of six mice per group, each dot is a single replicate and data are from a single experiment. *p < 0.05, **p < 0.01, ***p < 0.001 (one-way ANOVA).

As described above, the GIL peptide in the peptide pool was able to induce GIL-specific T cell responses when mixed with WIV (Figure 5A), while IFA-adjuvanted GIL peptide induced significantly lower T cell responses. The modified G1 peptide was able to induce some GIL-specific responses, regardless of adjuvant; however, these responses were not statistically higher than the negative control group. The G1 peptide adjuvanted with either WIV or IFA did induce a G1-specific T cell response, indicating that while the modified peptide was immunogenic in combination with an adjuvant, it failed to induce high responses that were cross-reactive with the wild-type (WT) analog.

The WT FMY peptide was able to induce FMY-specific T cell responses in combination with either WIV or IFA (Figure 5B). Interestingly, the modified F5 peptide was able to induce significantly higher FMY-specific responses compared to the WT FMY peptide when adjuvanted with WIV. F5 peptide adjuvanted with IFA did not show such an increase, indicating that WIV is a more potent adjuvant than IFA for the F5 peptide. This difference was also observed for the F5-specific responses; F5 peptide induced significantly higher F5-specific T cell responses when adjuvanted with WIV than with IFA.

Finally, the NML peptide and the modified N53 were unable to induce any significant T cell responses, regardless of adjuvant (Figure 5C). IFA-adjuvanted peptides showed incidental T cell responses in some animals, but these did not significantly differ from the WIV groups.

The individual peptides in both WT and modified pools did not show significant association with the WIV particles, similar to the previous observations with the GIL peptide alone (Table S1 in Supplementary Material). Thus, it is unlikely that differences in induced immune responses by the peptide vaccines were caused by differences in association between peptide and WIV.

In conclusion, two distinct effects were observed. First, we observed that WIV has an adjuvating effect on several peptides, including GIL, G1, FMY, and F5, indicating that WIV can also have an immunostimulating effect when combined with peptides other than GIL. Second, these data show that the F5 peptide was able to induce T cells which are cross-reactive with their wild-type analogs, which was also found in a previous study by Rosendahl Huber et al. (11).

## Discussion

Short peptides covering conserved internal influenza T cell epitopes can induce cross-reactive T cell responses. However, the peptides themselves are not immunogenic enough to elicit strong T cell responses. In recent years, multiple adjuvants have been utilized to improve the immunogenicity of such peptides, including Pam2Cys and CpG-ODN (20, 21), delivery systems, such as liposomes and virosomes (22, 23) and combinations thereof (14, 24). However, such combinations often require complex multi-step formulation procedures, which are difficult to produce on a large scale. In addition, antigen encapsulation and/or adsorption is sometimes very low, hampering large-scale production of such formulations. Furthermore, some of the adjuvants have not received an approval for human use, which hinders the evaluation of such vaccine formulations in human trials.

In this study, we improved the immunogenicity of influenza T cell epitopes by simply adding WIV to the peptides. We showed that the combination of WIV and GIL peptide induced significantly higher T cell responses than the individual components. By screening various concentrations of GIL and WIV, we found that both GIL and WIV synergistically contributed to the GIL-specific T cell response. This confirms earlier findings that WIV alone is able to induce influenza-specific T cell responses in mice (25, 26).

While these results showed that peptide and WIV together elicit strong T cell responses, the exact mode of action of the observed adjuvant effect of WIV is still unclear. Similar particulate delivery systems, such as virosomes, facilitate the escape of peptide antigens from the endosomal compartment into the cytosol, enabling processing of the peptide in the MHC-I pathway (14). This endosomal escape is mediated by the membrane fusion capabilities of influenza surface proteins. We showed, however, that endosomal escape due to membrane fusion of the WIV particle is most likely not the mechanism of action, since inactivation of the membrane fusion capabilities of WIV had no effect on its adjuvant activity. In addition, we showed a lack of physical association between WIV and peptide, which indicates that it is unlikely that WIV functions as a particulate carrier system. This may be confirmed in a future study by conjugating the peptide with WIV and determine the immunogenicity of this construct. It is possible that the GIL peptides are cross-presented by the dendritic cells (DCs) *via* the vacuolar pathway, which does not require endosomal escape of the antigen, but does require the DCs to display a mature and activated phenotype. To acquire this mature phenotype, interaction with PAMPs by pattern recognition receptors such as TLRs on the DC is critical. It is thus most likely that the presence of viral ssRNA in WIV provides strong TLR7 signaling (8), which activates the DCs and enables subsequent induction of T cell responses. Indeed, co-localization of the WIV particles with the peptide antigen was necessary to increase T cell responses; without WIV, T cell responses were low. This supports the hypothesis that local maturation of DCs through TLR7 activation might be required for efficient cross presentation of the peptide antigen and subsequent T cell induction. Stoel et al. confirmed that immature murine DCs matured after exposure to WIV; CD40, CD80, and CD86 surface expression increased significantly after 24 h (27). Tapia-Calle et al. found similar surface expression patterns in human DCs, and additionally showed that IRF7, STAT1, and MyD88, which all play a role in the TLR7 signaling pathway, were significantly increased in DCs upon stimulation with WIV (28).

Another contributing factor for the adjuvant effect of WIV might be related to an increased infiltration of inflammatory cells at the site of injection. Particulates such as WIV can activate innate cells, which is accompanied by the release of chemokines and cytokines (29). Particulate adjuvants can also activate NALP3 inflammasomes, which might enhance innate and cellular immunity (30). Inflammasomes are also known to be activated upon influenza virus infection (31). Whether the TLR7 pathway or the inflammasome pathway contributes to the adjuvanticity of WIV could be investigated in a future study using TLR7^−/−^ and ASC^−/−^ knockout mice, respectively.

In addition, WIV contains multiple other T cell antigens, which may induce CD4^+^ T helper cells, that in turn can aid CD8^+^ CTL priming (32). In addition to studies with the GIL peptide, we combined WIV with a mixture of dominant and subdominant epitopes, either in wild-type or CPL form. CPLs were selected based on their improved binding affinity on MHC-I molecules (19), which generally enhances immunogenicity (11). WIV was able to improve the homologous T cell responses of both GIL and G1 peptides. However, the modified G1 peptide did not induce higher T cell responses against the wild-type GIL epitope. By contrast, the modified F5 peptide was able to induce significantly higher FMY-specific T cell responses compared to WT FMY peptide in the presence of WIV. T cell responses against NML or N53 were almost not detectable; it is likely that the subdominant nature of the peptide was the cause of this. For the peptides GIL and F5, WIV might be able to replace IFA, which is commonly used as an adjuvant for peptide antigens.

The peptides used in this study are all HLA-A2.1 binding peptides, which is one of the most frequently occurring alleles in the Caucasian population. However, allele frequencies differ between ethnicities, and thus multiple peptide covering multiple alleles should be combined to ensure complete coverage in the human population. Such approaches using peptide pools have already reached phase IIb clinical studies (33–36), with positive results. These T cell-inducing influenza vaccines would be ideal to reduce the morbidity and mortality in vulnerable people exposed to highly pathogenic influenza strains in the event of a pandemic, when a strain-specific vaccine is not yet available.

A few comparable studies with HLA-A2.1 influenza peptides have been conducted previously in the literature. Recently, Herrera-Rodriguez et al. showed that a peptide pool consisting of four modified peptides adjuvanted with ISA-51 induced potent T cell responses in mice (37). These T cell responses were able to reduce weight loss and mortality in H1N1pdm-infected HLA-A2.1 transgenic mice. Our group previously showed that a single T cell peptide delivered by CpG-adjuvanted virosomes was also able to reduce weight loss in H3N2-infected HLA-A2.1 transgenic mice (14). Both these studies showed that vaccinated mice had approximately 200 IFN-γ-producing spot-forming units (SFU)/10^6^ cells, whereas our current study shows that mice vaccinated with (modified) peptides adjuvanted with WIV reach about 400–900 IFN-γ-producing SFU/10^6^ cells. This may be an indication that our WIV-adjuvanted peptides also offer some protection to heterologous influenza infection in mice.

Our current study is the first to show the adjuvant effect of WIV on peptide-induced CD8^+^ T cell responses. Earlier it was shown that WIV can also boost humoral responses; Babb et al. showed that gamma-irradiated WIV was able to boost specific IgG and IgG2c responses to a gamma-irradiated SFV vaccine (10), but did not report any effect on T cell responses. Our data show that T cell responses are also boosted by WIV. Future studies are required to determine the minimum amount of WIV required to induce this adjuvant effect, since the concentration of WIV used in this study are higher than the dose required for a standard influenza vaccination. Taken together, these data show that inactivated whole influenza virus is an effective adjuvant for CD8^+^ T cell peptide antigens, which is easy to formulate and has a long track record with regard to usage and safety.

## Ethics Statement

Animal studies were conducted according to the guidelines provided by the Dutch Animal Protection Act, and were approved by the Committee for Animal Experimentation (DEC) of the PD-Alt campus (Bilthoven, The Netherlands).

## Author Contributions

PS and SRH have designed and performed the experiment, as well as written the manuscript. G-JW, RJ, and MH have assisted with the immunological experiments. ES, WJ, JJ, JB, GK and J-PA have assisted with the experimental design, and assisted writing and reviewing the manuscript.

## Conflict of Interest Statement

PS, G-JW, GK, and J-PA are or were employed by Intravacc. ES is owner of the company Virtuvax. All other authors declare no competing interests.

## Abbreviations

WIV, whole-inactivated influenza virus; CTLs, cytotoxic CD8^+^ T cells; APCs, antigen-presenting cells; PAMPs, pathogen-associated molecular patterns; IFA, incomplete Freund’s adjuvant; CPLs, chemically altered peptide ligands; DCs, dendritic cells; GIL, GILGFVFTL (M1_58–66_) peptide; FMY, FMYSDFHFI (PA_46–54_) peptide; NML, NMLSTVLGV (PB1_413–421_) peptide; SFU, spot-forming units.
