# Supplementary material for: Whole-Inactivated Influenza Virus Is a Potent Adjuvant for Influenza Peptides Containing CD8+ T Cell Epitopes
Source: Front Immunol. 2018 Mar 14;9:525. doi: 10.3389/fimmu.2018.00525 (PMC5861146; doi:10.3389/fimmu.2018.00525)
Supplement: Supplementary file 1 [file data_sheet_1.docx]

Supplementary Material

Whole-inactivated Influenza Virus Is a Potent Adjuvant for Influenza Peptides Containing CD8^+^ T Cell Epitopes

Peter C. Soema ^1,2†^, Sietske K. Rosendahl Huber ^3†^, Geert-Jan Willems ^1^, Ronald Jacobi ^3^, Marion Hendriks ^3^, Ernst Soethout ^1‡^, Wim Jiskoot ^2^, Jørgen de Jonge ^3^, Josine van Beek ^3^, Gideon F. A. Kersten ^1,2^, Jean-Pierre Amorij ^1^

^†^Authors contributed equally

^1^ Intravacc (Institute for Translational Vaccinology), Bilthoven, The Netherlands

^2^ Division of Drug Delivery Technology, Cluster BioTherapeutics, Leiden Academic Centre for Drug Research (LACDR), Leiden University, Leiden, The Netherlands

^3^ Centre for Infectious Disease Control Netherlands, National Institute for Public Health and the Environment (RIVM), Bilthoven, The Netherlands

^‡^ Current affiliation: Virtuvax, Odijk, The Netherlands

* Correspondence:

Dr. Peter Soema

Intravacc

Antonie van Leeuwenhoeklaan 9, 3721 MA Bilthoven, The Netherlands

[Peter.soema@intravacc.nl](mailto:Peter.soema@intravacc.nl)

# Supplementary Figures and Tables

## Supplementary Figures


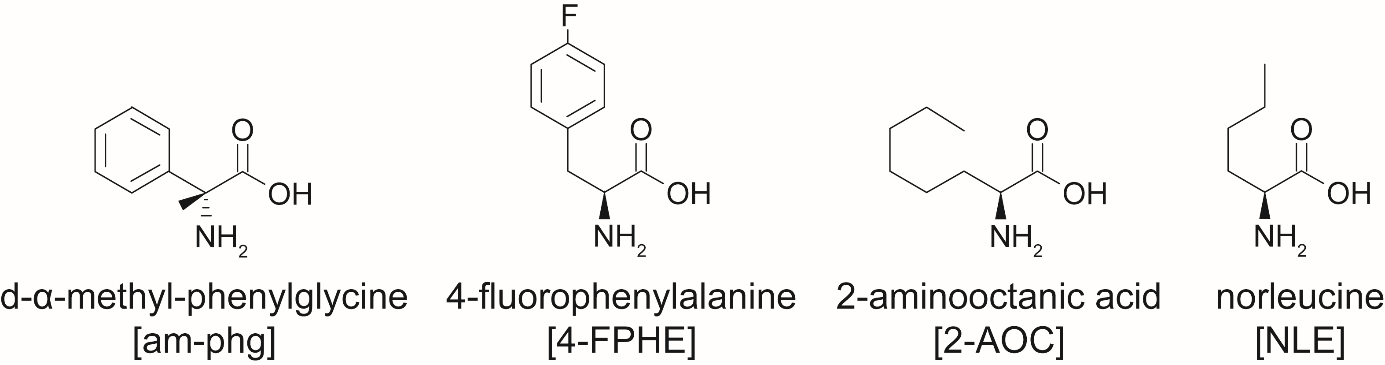


**Supplementary Figure 1. Nonproteogenic synthetic amino acids used for peptide modification.** The four nonproteogenic synthetic amino acids were introduced either in GILGFVFTL, FMYSDFHFI or NMLSTVLGV peptides, resulting in modified [am-phg]ILGFVFTL, [4-FPHE]MYSDFHF[2-AOC] and N[NLE]LSTVLGV peptides.


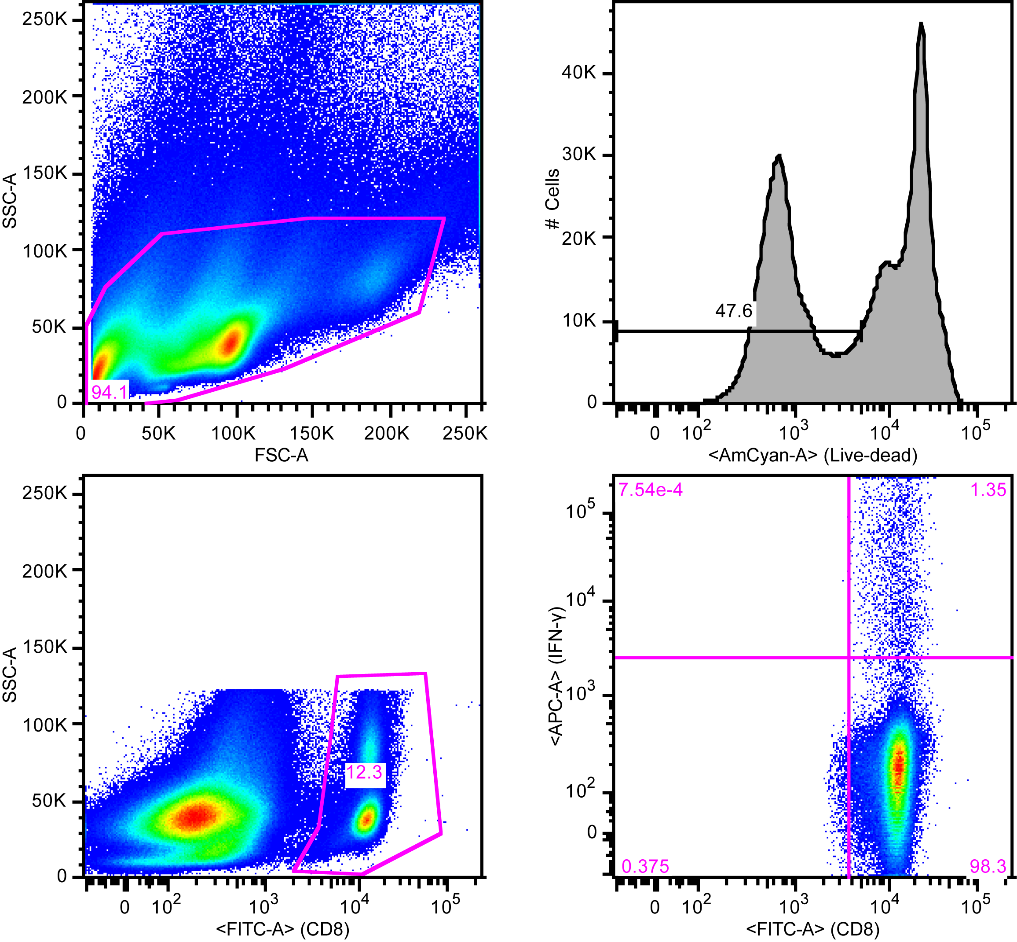


**Supplementary Figure 2. Gating strategy of CD8^+^ IFN-γ^+^ T cells in splenocytes.** An example of the gating strategy. The lymphocyte population was first gated (upper left). From this population, all live cells were selected (upper right). Subsequently, CD8^+^ cells were gated (lower left), after which a quadrant gate was created to select for CD8^+^ IFN-γ^+^ cells (lower right).

## Supplementary Tables

**Table S1. Association of peptides with WIV.** Peptides were admixed with WIV (in similar concentrations as used in vivo) and subsequently separated by ultracentrifugation. The residual, unassociated peptide in the supernatant was quantified using by mass spectrometry. Data are shown as mean ± SD from three individual experiments.

| **Peptide** | **Unassociated peptide (%)** |
| --- | --- |
| **GIL** | 87 ± 18 |
| **FMY** | 81 ± 22 |
| **NML** | 115 ± 21 |
| **G1** | 138 ± 34 |
| **F5** | 96 ± 8 |
| **N53** | 99 ± 12 |
